# Inefficiency in Delivery of General Surgery to Black Patients: A National Inpatient Sample Study

**DOI:** 10.1055/s-0043-1777811

**Published:** 2023-12-19

**Authors:** John N. Bliton

**Affiliations:** 1 Department of Surgery, Jamaica Hospital Medical Center, Queens, New York

**Keywords:** general surgery, disparities, minorities, treatment disparities, discrimination

## Abstract

**Background** Racial disparities in outcomes among patients in the United States are widely recognized, but disparities in treatment are less commonly understood. This study is intended to identify treatment disparities in delivery of surgery and time to surgery for diagnoses managed by general surgeons—appendicitis, cholecystitis, gallstone pancreatitis, abdominal wall hernias, intestinal obstructions, and viscus perforations.

**Methods** The National Inpatient Sample (NIS) was used to estimate and analyze disparities in delivery of surgery, type of surgery received, and timing of surgery. Age-adjusted means were compared by race/ethnicity and trends in treatment disparities were evaluated from 1993 to 2017. Linear modeling was used to measure trends in treatment and outcome disparities over time. Mediation analysis was performed to estimate contributions of all available factors to treatment differences. Relationships between treatment disparities and disparities in mortality and length of stay were similarly evaluated.

**Results** Black patients were less likely to receive surgery for appendicitis, cholecystitis, pancreatitis, and hernias, and more likely to receive surgery for obstructions and perforations. Black patients experienced longer wait times prior to surgery, by 0.15 to 1.9 days, depending on the diagnosis. Mediation analysis demonstrated that these disparities are not attributable to the patient factors available in the NIS, and provided some insight into potential contributors to the observed disparities, such as hospital factors and socioeconomic factors.

**Conclusion** Treatment disparities are present even with common indications for surgery, such as appendicitis, cholecystitis, and gallstone pancreatitis. Black patients are less likely to receive surgery with these diagnoses and must wait longer for surgery if it is performed. Surgeons should plan institution-level interventions to measure, explain, and potentially correct treatment disparities.

Black patients in the United States face disadvantages in medical treatment and outcomes that have persisted across generations, including among surgical patients. 1 Some progress has been observed in the research on racial differences in medicine, especially considering that early articles were more akin to racist pseudoscience than valid studies. 2 More recent publications document and lament worse outcomes among minority patients, 3 but frequently fall short of being able to provide any clear “next steps” to correct disparities. Accordingly, authors have emphasized the need to move from such purely descriptive studies of outcome disparities toward action-guiding science. 4

Although the most direct way to meet this goal would be to identify interventions that correct disparities, 5 describing treatment disparities may be a valuable step forward. 6 7 8 9 10 11 12 Identification of treatment disparities could help physicians ameliorate disparities through direct action by changing their own behavior or institutional priorities. For example, surgeons could improve the observed disparity in delivery of inpatient cholecystectomy after presentation for gallstone pancreatitis 13 by ensuring they close any such gap at their own institution. Thus, this project seeks to measure treatment disparities in management of six acute care surgery diagnoses in the United States. Furthermore, the project performs a mediation analysis that identifies potential contributors to both treatment and outcome disparities (in mortality and length of stay [LOS]) and allows for comparisons among these diagnoses.

The National Inpatient Sample (NIS), years 1993 to 2017, was used to perform this analysis and provide a preliminary assessment of these questions. 14 The diagnoses explored are common indications for performing surgery before a patient is discharged from the hospital: appendicitis, cholecystitis, gallstone pancreatitis, hernias, bowel obstructions, and viscus perforations. By estimating treatment disparities (and ruling out many alternative explanations for such gaps), 15 it may be possible to encourage surgeons to improve care of minority patients.

## Materials and Methods

### Sample and Variable Description

The NIS is a publicly available database that provides a large cohort of discharges with diagnoses of common urgent or emergent indications for abdominal surgery in the United States. The methods of NIS sampling have changed over time—prior to 2012, it used a 20% stratified random sample of inpatient discharges of all hospitals from 48 U.S. states. Most recently, the NIS uses discharge records from the Healthcare Quality Utilization Project-participating hospitals. This data is shared by hospitals with the Agency for Healthcare Research and Quality (part of the U.S. Department of Health and Human Services) and statistical weights are included to ensure that analyses produce accurate estimates of the overall public health reality in the U.S. 14 The variables available in the sample change over time—for example, in 2012, hospital identifiers were changed such that hospital-level statistics could no longer be calculated, and in 2015, a transition was made from International Classification of Diseases, Ninth Revision (ICD-9) to ICD-10 codes. In this study, means and mediation analyses (described below) were calculated based on years 2002 to 2011 when the data set is most complete, as well as for 2016 to 2017 to create a more recent estimate. Trends over time in disparities were calculated with a more limited covariate adjustment based on years 1993 to 2017. The available variables expanded substantially and became more useful in 1993.

Inclusion criteria were primary diagnostic codes consistent with the diagnoses under study. ICD-9 and ICD-10 codes for appendicitis, cholecystitis, gallstone pancreatitis (based on pancreatitis with concomitant gallstones among secondary diagnostic codes in ICD-9), abdominal wall hernias, bowel obstructions, and viscus perforations are listed in Supplementary Appendix 1 (available in the online version), as well as extensive classifications made based on these codes. The only exclusion criterion was the unavailability of race/ethnicity variables. Procedures were extracted and classified based on how definitive and invasive the surgeries were, with specific categorizations varying depending on diagnosis (details are in Supplementary Appendix 1 , available in the online version). A separate classification was made for temporizing procedures, such as percutaneous cholecystostomies in cholecystitis or endoscopic retrograde cholangiopancreatography in gallstone pancreatitis. Race/ethnicity was defined by classifying those listed with Hispanic ethnicity as Latinx race/ethnicity, and otherwise using the race variable itself. For readability and to avoid extensive potential confounding by immigrant status, emphasis is placed on black/white comparisons. Variables extracted aside from age and race/ethnicity included socioeconomic/logistical factors (ZIP income quartile, insurance type, weekend admission, and elective vs. nonelective admission), treatment factors (whether surgery was performed, time to surgery, surgery type), medical factors (comorbidity score, diagnosis subgroups based on mortality, and whether unusual steps were required during surgery), hospital factors (size, ownership, region, mean LOS, and operative rate), and outcomes (LOS and inpatient mortality).

Some variables listed above require further comment. For years 1993 to 2011, hospital IDs were used to calculate hospital-level operative rates and mean LOS. Time to surgery and postoperative LOS were calculated based on the first procedure day. Patients with disease-specific surgery, such as an appendectomy in appendicitis, prior to admission, were excluded. For each diagnosis, mortality was calculated by diagnostic ICD code, and then the codes were empirically classified into three diagnosis subgroups based on the observed mortality. Also for each diagnosis, three categorizations were created for types of surgery received. For example, in appendicitis, appendectomies were in the least invasive category, the intermediate category included appendectomies with lysis of adhesions, and the most invasive category included additional intestinal resections. In hernias, the first category includes procedures with mesh repairs, and the last category includes intestinal resections, with the remainder falling in the intermediate category. Of note, this variable is treated as nominal categorical and nonordinal in all analyses, since the categorizations are not truly 3 points on a spectrum. Excess mortality risk associated with each comorbidity was calculated on a per-diagnosis basis, then combined to create a crude comorbidity risk score. The ICD codes are included in Supplementary Appendix 1 , available in the online version for all ICD-based variables.

### Statistical Analyses

All analyses were conducted using the “survey” package in R to account for both clustering and stratification. The TRENDWT variable was used for the analyses of trends over time and the DISCWT variable was used for all other analyses. 16 Summary statistics were calculated for each variable by race/ethnicity. Confidence intervals were calculated to compare treatment factors and outcomes by race/ethnicity with adjustment for age. Trends in disparities over time were visualized based on age-adjusted means for years 1992 to 2017, exclusive of 2015 since NIS methods were significantly changed mid-year. Simple regression was performed to determine whether disparities were increasing or decreasing over time. LOS and postoperative LOS were compared with and without standardization for treatment factors (time to surgery and surgery type).

For years 2002 to 2011, multiple mediation analysis was performed via the difference of coefficients method to estimate contributions of all previously listed medical, treatment, hospital, and socioeconomic factors to outcome disparities. 17 18 Mediation analysis of the NIS is limited by the inherent disadvantages of using an administrative database; all potential relationships established would require more direct confirmation than can be performed with the database alone. However, mediation analysis allows for preliminary assessment of the statistical contribution of each of these categories of factors by including and excluding them from the regression models and observing changes in the race-related coefficient. Put another way, mediation analysis involves breaking down a “total effect”—in this case, the association between race and the outcome—into “specific indirect effects” and “direct effects.” The specific indirect effects are the portions of the total effect that are statistically attributable to variables in the model—in this case, medical, treatment, hospital, or socioeconomic factors. Lastly, the “direct” effect is the portion of the disparity that remains unexplained after inclusion of the factors in the model. 19 In seeking to understand disparities, it is inappropriate to assume that any unexplained relationship with race is truly “direct,” since race is a social construct rather than a scientifically meaningful biological category.

Age and year were excluded from the mediation analysis by adjusting for them in all models being compared. Three sensitivity analyses were performed; the mediation analysis was repeated excluding all elective admissions, including only years 2016 to 2017, and including additional variables (whether the patient left against medical advice and with individual comorbidity variables rather than the comorbidity score).

## Results

### Sample Characteristics

For years 2002 to 2011, 1,679,073 discharges were included—345,101 with appendicitis, 562,371 with cholecystitis, 107,082 with gallstone pancreatitis, 249,199 with hernias, 376,078 with obstructions, and 39,242 with perforations ( Table 1 ). For trend analyses, a total of 3,848,533 discharges from years 1993 to 2017 were included. Black patients composed between 6.9 and 11.6% of the included patients for each diagnosis. White patients represented between 66.0 and 76% depending on the diagnosis. White patients were older, with mean ages ranging from 42 to 67 depending on the diagnosis, whereas the range was 38 to 60 among black patients. The difference between the black and white mean ages ranged from 4 to 8 years depending on the diagnosis. Black patients more frequently had Medicaid (i.e., 18.7% vs. 6.7% for appendicitis) or no insurance (17.6% vs. 9.0%) and were the more likely to live in ZIP codes of the lowest income quartile (50.8% vs. 17.6%). Black patients were less likely to be classified as having been admitted electively (i.e., 34.0% vs. 48.1% for hernias). Differences in the proportions of patients from each racial/ethnic group in the sample were also observed, which is expected given differences in risk factors, such as fertility and obesity rates in cholecystitis.

**Table 1:** Descriptive statistics

			Appendicitis		Cholecystitis		Gallstone pancreatitis		Hernias		Obstructions		Perforations	
			Black	White	Black	White	Black	White	Black	White	Black	White	Black	White
General	*N*		23,669	230,880	52,327	374,942	11,784	70,692	27,669	186,669	43,528	286,780	4,551	29,817
	Percent of all racial/ethnic groups		6.9%	66.9%	9.3%	66.7%	11.0%	66.0%	11.1%	74.9%	11.6%	76.3%	11.6%	76.0%
	Mean age (y)		38.2	42.3	49.6	57.0	53.1	60.4	54.9	62.9	59.3	65.7	54.7	65.1
	Age	< 40 years old	57.7%	47.5%	33.0%	21.5%	22.8%	15.8%	18.7%	8.4%	13.2%	7.7%	18.8%	8.6%
		40–54	26.0%	27.0%	28.6%	23.4%	32.3%	21.6%	33.2%	24.0%	27.2%	17.9%	34.5%	19.5%
		55–69	11.6%	17.1%	21.7%	24.7%	25.4%	26.1%	27.4%	31.3%	28.4%	27.5%	25.7%	26.5%
		70–89	4.5%	8.0%	15.6%	28.3%	17.9%	33.0%	19.3%	33.7%	28.3%	42.4%	18.8%	40.3%
		> 89	0.2%	0.3%	1.1%	2.2%	1.5%	3.4%	1.4%	2.5%	2.9%	4.5%	2.2%	5.0%
Medical*	Mean comorbidity count		0.31	0.28	0.71	0.67	0.93	0.88	0.71	0.72	0.99	0.98	0.89	1.12
	Percent female		46.6%	47.1%	75.7%	61.6%	62.4%	56.8%	60.1%	55.9%	60.6%	58.7%	41.5%	56.6%
	Diagnosis-based mortality risk *	Lowest	66.4%	66.4%	76.9%	73.9%	3.5%	2.2%	39.7%	47.0%	1.6%	1.0%	61.5%	51.7%
		Intermediate	3.7%	2.8%	5.3%	6.0%	43.8%	49.9%	59.0%	51.6%	93.5%	94.6%	8.9%	8.3%
		Highest	29.9%	30.8%	17.8%	20.1%	52.7%	48.0%	1.3%	1.4%	4.9%	4.4%	29.7%	40.0%
	Surgical difficulty *	Intermediate category	40.4%	37.4%	13.8%	13.3%	1.6%	1.6%	23.5%	23.5%	4.1%	2.8%	33.4%	40.4%
		Most invasive	3.1%	2.3%	0.0%	0.1%	0.7%	0.8%	4.4%	4.7%	13.7%	12.8%	0.1%	0.2%
		Least invasive, most definitive	56.5%	60.3%	86.1%	86.6%	97.8%	97.7%	72.1%	71.8%	82.2%	84.4%	66.5%	59.4%
Socioeconomic	Payer	Medicaid	18.7%	6.7%	20.8%	7.6%	20.9%	7.3%	17.4%	6.2%	13.7%	4.5%	16.7%	6.0%
		Medicare	10.1%	13.6%	29.6%	40.3%	34.6%	46.6%	36.6%	50.0%	46.7%	59.0%	33.0%	55.1%
		No charge	1.9%	0.9%	1.3%	0.6%	1.3%	0.6%	1.4%	0.4%	0.9%	0.3%	2.6%	0.7%
		Other	5.1%	3.5%	3.3%	2.6%	3.9%	2.7%	4.5%	3.3%	3.5%	1.9%	6.1%	2.8%
		Private	46.1%	66.1%	35.1%	42.5%	27.8%	36.3%	31.4%	36.5%	28.4%	31.7%	22.9%	28.9%
		Self-pay	17.6%	9.0%	9.6%	6.2%	11.1%	6.3%	8.4%	3.4%	6.7%	2.6%	18.1%	6.2%
		Unavailable	0.4%	0.2%	0.3%	0.2%	0.3%	0.2%	0.5%	0.2%	0.2%	0.1%	0.4%	0.2%
	ZIP income quartile	Lowest	40.8%	17.6%	45.9%	22.7%	49.6%	22.9%	48.9%	21.6%	45.9%	20.5%	53.5%	23.2%
		Second	22.3%	23.0%	22.7%	25.7%	21.8%	26.2%	21.5%	25.9%	22.1%	25.4%	21.2%	27.0%
		Third	19.1%	25.8%	17.4%	25.0%	15.7%	24.8%	16.1%	25.5%	16.8%	25.3%	14.2%	25.2%
		Highest	14.9%	31.5%	11.6%	24.4%	10.3%	23.9%	10.6%	24.9%	12.5%	26.8%	7.8%	22.4%
		Unavailable	2.9%	2.1%	2.4%	2.2%	2.7%	2.2%	2.9%	2.2%	2.6%	2.0%	3.3%	2.3%
	Weekend admission		25.1%	23.9%	21.5%	20.6%	26.3%	26.2%	15.2%	11.8%	26.1%	25.3%	28.1%	25.2%
	Elective admission		3.4%	5.6%	14.8%	18.8%	4.5%	6.6%	34.0%	48.1%	4.8%	7.6%	3.6%	6.2%
	Percent who left against medical advice		0.5%	0.2%	0.9%	0.4%	2.5%	0.9%	0.8%	0.3%	1.9%	0.8%	0.8%	0.4%
Hospital	Ownership	For profit	13.8%	11.7%	14.9%	13.6%	14.0%	12.8%	11.8%	11.7%	13.0%	11.5%	12.6%	11.8%
		Government	10.9%	8.6%	10.8%	9.8%	12.0%	9.3%	11.3%	8.9%	11.6%	9.5%	12.0%	10.0%
		Nonprofit	41.4%	45.9%	40.0%	44.7%	38.9%	45.7%	39.6%	43.7%	42.9%	47.9%	37.5%	44.2%
		Unspecified private	33.9%	33.7%	34.3%	32.0%	35.1%	32.2%	37.3%	35.7%	32.4%	31.1%	38.0%	34.0%
	Size	Large	60.4%	57.4%	61.6%	58.8%	61.3%	57.6%	62.7%	61.2%	62.5%	58.1%	63.5%	59.5%
		Medium	27.3%	27.1%	26.8%	26.4%	26.8%	26.9%	25.9%	25.4%	25.6%	26.0%	26.5%	26.3%
		Small	11.8%	15.3%	11.2%	14.5%	11.3%	15.0%	10.8%	13.1%	11.4%	15.6%	9.4%	13.8%
		Unavailable	0.4%	0.3%	0.4%	0.3%	0.6%	0.4%	0.5%	0.3%	0.5%	0.3%	0.6%	0.3%
	Type	Rural	6.7%	14.4%	8.0%	16.3%	8.4%	15.3%	6.4%	12.2%	7.5%	15.8%	7.7%	15.6%
		Urban, nonteaching	38.8%	50.8%	41.4%	52.5%	37.0%	49.2%	33.2%	46.2%	36.6%	47.8%	34.3%	48.0%
		Urban, teaching	54.1%	34.6%	50.3%	30.9%	54.0%	35.0%	59.9%	41.3%	55.4%	36.0%	57.3%	36.1%
		Unavailable	0.4%	0.3%	0.4%	0.3%	0.6%	0.4%	0.5%	0.3%	0.5%	0.3%	0.6%	0.3%
	Delivery of surgery or drainage		96.9%	97.6%	88.5%	89.5%	60.5%	62.4%	87.6%	88.7%	44.0%	42.7%	89.6%	87.9%
	Hospital % black patients		18.9%	6.6%	22.3%	8.2%	28.7%	11.3%	24.5%	9.2%	27.3%	10.1%	33.1%	12.9%
	Per-hospital white-black operative rate difference		1.0%	0.5%	1.8%	1.5%	9.4%	8.9%	2.5%	2.8%	-2.4%	-3.1%	-4.2%	-5.0%

* Classifications of diagnostic codes by mortality risk, and procedural code by surgical difficulty, are provided in Supplementary Appendix 1 , available in the online version.

### Age-Adjusted Treatment and Outcome Disparities

**Fig. 1:**
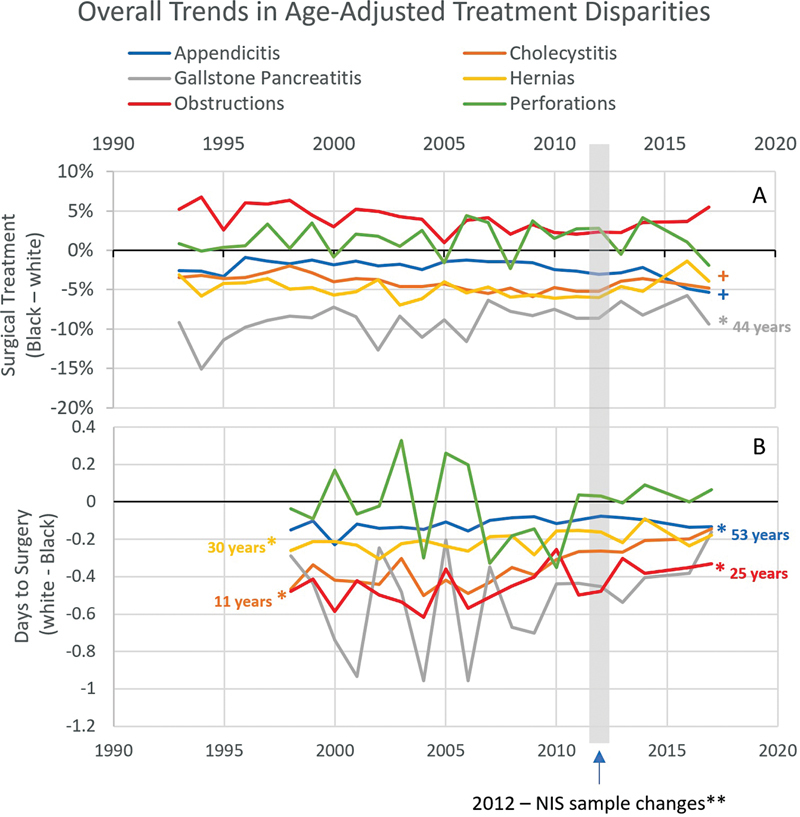
Trends over time in treatment and outcome disparities, age adjusted. ( **A** ) Percent of black patients who received surgery minus percent of white patients per year (negative numbers correspond to lower operative rates for black patients). ( **B** ) Mean difference, white minus black length of stay, in days. Day of surgery data was only available after 1997. *Difference is decreasing over time, *p* < 0.05, label includes years until closure of the gap based on the 2017 disparity and slope. **+** Difference is increasing over time, *p* < 0.05. **In 2012, the hospitals from which the National Inpatient Sample (NIS) sources their patient data changed.

Age-adjusted treatment and outcome differences were assessed to estimate the overall disparities which will be further investigated in the multivariable mediation analysis. Treatment differences were present in all diagnoses ( Table 2 ). Black patients were less likely to have surgery for appendicitis, cholecystitis, gallstone pancreatitis, and hernias. They were more likely to have surgery for perforations and obstructions. Black patients waited longer for surgery with every diagnosis. The shortest differences were for appendicitis and perforations (0.15 and 0.19 days, respectively), and the longest difference was for obstructions (1.9 days). Trends in treatment disparities are displayed in Fig. 1 for years 1993 to 2017; trends in outcome disparities are available in Supplementary Appendix 2 , available in the online version. In cholecystitis, the age-adjusted disparities appear to be improving over time.

**Table 2:** Age-adjusted treatment and outcome differences

		Race/Ethnicity							
		Black	SE	White	SE	White vs. black *p* -value	Latinx	API	Native/ other
Any surgery	Appendicitis	96.0%	0.2%	97.9%	0.1%	< 0.001	97.2%	97.0%	97.3%
	Cholecystitis	85.3%	0.3%	89.8%	0.2%	< 0.001	88.5%	88.0%	86.7%
	Gallstone pancreatitis	52.8%	0.9%	62.8%	0.5%	< 0.001	66.9%	62.4%	62.7%
	Hernias	87.0%	0.3%	91.4%	0.1%	< 0.001	87.6%	90.8%	88.9%
	Obstructions	45.3%	0.5%	41.9%	0.3%	<0 .001	35.5%	37.7%	41.6%
	Perforations	89.4%	0.6%	87.5%	0.3%	< 0.001	87.1%	88.5%	87.5%
Length of stay, d	Appendicitis	4.19	0.05	2.94	0.02	< 0.001	3.17	2.84	3.02
	Cholecystitis	5.27	0.06	4.01	0.03	< 0.001	4.46	4.23	4.31
	Gallstone pancreatitis	7.03	0.09	6.36	0.06	< 0.001	6.80	6.79	6.91
	Hernias	5.60	0.06	4.43	0.03	< 0.001	4.52	4.42	4.48
	Obstructions	8.40	0.09	6.50	0.03	< 0.001	6.41	6.18	6.52
	Perforations	12.46	0.24	11.63	0.12	< 0.001	12.36	10.83	11.15
Mortality	Appendicitis	0.6%	0.1%	0.2%	0.0%	< 0.001	0.2%	0.1%	0.3%
	Cholecystitis	0.9%	0.1%	0.6%	0.0%	< 0.001	0.7%	0.5%	0.6%
	Gallstone pancreatitis	1.4%	0.1%	1.3%	0.1%	0.287	1.4%	1.7%	1.2%
	Hernias	1.8%	0.1%	1.2%	0.0%	< 0.001	1.2%	0.8%	1.4%
	Obstructions	4.0%	0.1%	2.8%	0.1%	< 0.001	3.0%	2.4%	2.2%
	Perforations	14.1%	0.6%	14.4%	0.3%	0.664	14.8%	11.5%	15.4%
Laparoscopic surgery	Appendicitis	50.0%	1.1%	55.8%	0.8%	< 0.001	54.7%	55.0%	52.8%
	Cholecystitis	78.9%	0.5%	83.7%	0.3%	< 0.001	83.8%	82.2%	82.9%
	Gallstone pancreatitis	77.2%	1.1%	77.3%	0.5%	0.747	79.7%	69.0%	77.1%
	Hernias	64.7%	0.6%	72.8%	0.3%	< 0.001	69.5%	68.3%	70.8%
	Obstructions	63.5%	0.7%	59.1%	0.5%	< 0.001	55.9%	54.9%	54.7%
	Perforations	67.1%	1.0%	63.5%	0.4%	< 0.001	64.1%	73.1%	65.4%
Drainage/ERCP/diversion only	Appendicitis	3.7%	0.2%	2.0%	0.1%	< 0.001	2.1%	2.6%	2.3%
	Cholecystitis	3.0%	0.1%	1.8%	0.1%	< 0.001	1.9%	1.7%	2.6%
	Gallstone pancreatitis	42.4%	1.2%	44.7%	0.7%	< 0.001	44.3%	54.5%	43.1%
	Hernias	35.7%	0.6%	28.1%	0.3%	< 0.001	31.4%	31.1%	30.2%
	Obstructions	37.8%	1.1%	39.2%	0.7%	< 0.001	39.8%	46.5%	42.1%
	Perforations	21%	1%	21%	0%	0.284	21%	20%	22%
Time to surgery, days	Appendicitis	0.40	0.01	0.25	0.00	< 0.001	0.31	0.24	0.27
	Cholecystitis	1.94	0.04	1.40	0.02	< 0.001	1.63	1.36	1.57
	Gallstone pancreatitis	3.76	0.07	3.23	0.04	< 0.001	3.39	3.09	3.47
	Hernias	0.87	0.02	0.49	0.01	< 0.001	0.64	0.60	0.60
	Obstructions	2.40	0.05	1.82	0.02	< 0.001	2.13	1.61	1.71
	Perforations	0.94	0.07	0.75	0.02	< 0.001	0.96	0.65	0.70

Abbreviations: API, Asian/Pacific Islander; ERCP, endoscopic retrograde cholangiopancreatography; SE, standard error.

**Fig. 2:**
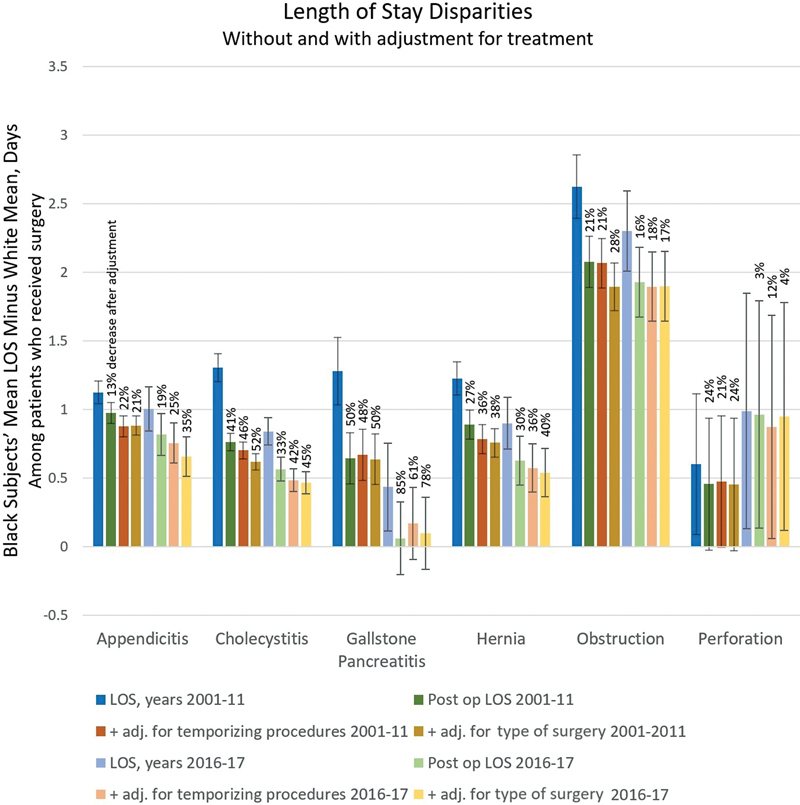
Age-adjusted length of stay disparity without and with adjustment for treatment factors, 2001–2011 and 2016–2017. The disparities in days between mean lengths of stay for black and white patients are displayed with 95% confidence intervals. The data labels are the percent change with the listed adjustments for treatment. Postop length of stay disparities are displayed without further adjustment, with standardization for receipt of drainage, and with standardization for receipt of more or less invasive or definitive surgery (procedural details are in Supplementary Appendix 1 , available in the online version). LOS, length of stay; Postop, postoperative; adj, adjusted.

As seen in Table 2 , outcome disparities were noted for all diagnoses. Fig. 2 demonstrates the association between treatment factors and LOS without multivariable adjustment. Differences in treatment accounted for significant portions of disparities in LOS among patients who received surgery (for 2001–2011, appendicitis: 21%, cholecystitis: 52%, gallstone pancreatitis: 50%, hernias: 38%, obstructions: 28%, perforations: 24%). For years 2016 to 2017, the entire disparity in LOS for gallstone pancreatitis could be explained by the disparate wait time between admission and surgery. The type of procedure performed—such as minimally invasive surgery as compared to open surgery—also had a small contribution in some cases.

### Mediation Analysis

**Fig. 3:**
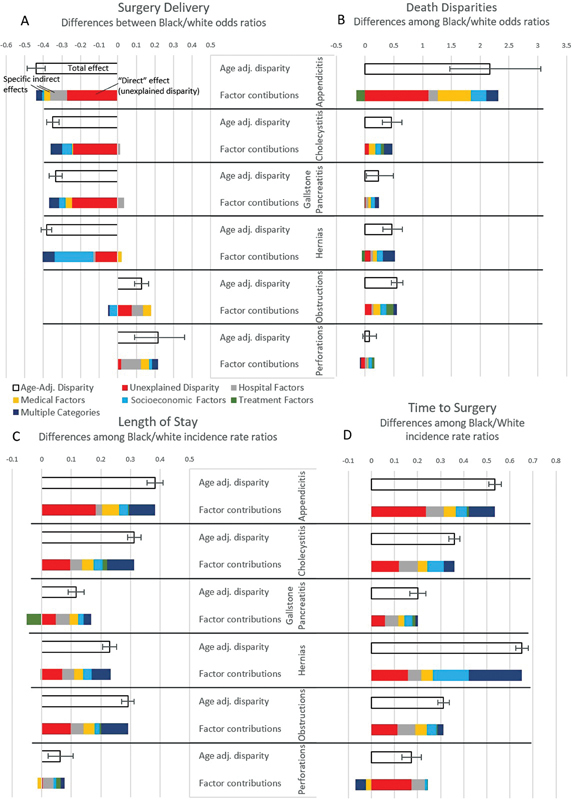
Multiple mediation analysis. Disparities based on coefficients from generalized linear models, or “total effects” in mediation analysis nomenclature, are displayed with black outlines. The adjusted specific indirect effects for groups of variables are displayed—these correspond to the portion of the disparity statistically independently associated with the factor group. They are calculated by comparing coefficients associated with race with and without adjustments for the factors in the group. The “direct” effect is the residual relationship between race and the outcome after adjusting for all other factors, or the disparity not explained by other factors in the model. Demographic factors: ZIP income quartile, insurance type, weekend admission, and elective vs. nonelective admission. Treatment factors: whether surgery was performed, time to surgery, and surgery type. Medical factors: comorbidities, diagnosis subgroups based on mortality, and necessity of unusual surgical steps. Hospital factors: size, ownership, region, mean length of stay (LOS) (for models LOS and surgery day), and operative rate (for mortality and surgery delivery).

Fig. 3 shows the results of the mediation analysis and provides a preliminary conceptual sketch of potential contributors to the disparities. Age and year were adjusted for prior to the mediation analysis; the statistical relationships with all other variable groups are presented in the figure. For example, consider the LOS disparity of 1.28 days in cholecystitis ( Table 2 ). This disparity is defined by an incidence rate ratio of 0.31 (this is the total effect, reflecting a ratio in the probability of discharge per day), and all but one-third of this disparity is attributable to factors in the model (the direct effect or unexplained disparity, represented by the red box, is 0.12). Part of the disparity is alternatively attributable to multiple factors (dark blue box, contribution: 0.09), the most significant independently contributing category is medical factors (yellow box, 0.04), and there is an independent contribution of treatment disparities (green box, 0.02). It should be noted that these differences represent within-hospital disparities, since hospital mean LOS was adjusted for (and is represented by the gray box). Prior to adjustment for medical, hospital, and socioeconomic factors, the influence of treatment disparities on LOS seemed more pronounced (as seen in Fig. 2 ).

To summarize across diagnoses, differences in delivery of surgery for appendicitis, cholecystitis, gallstone pancreatitis, and hernias are only partially attributable to differences in medical factors (comorbidities, diagnosis subgroups based on mortality, and whether unusual steps were required during surgery) or socioeconomic/logistical factors (ZIP income quartile, insurance type, weekend admission, and elective admission). In contrast, the higher rates of surgery for perforations among black patients were attributable to other factors in the model. Also, many of the measured disparities in outcomes were accounted for by other variables in the model, with the notable exception of mortality after appendicitis. Medical, hospital, and socioeconomic factors appear to independently explain portions of most disparities. For inpatient mortality, although substantial disparities are identified, they do not appear to be attributable to differential treatment. Small potential independent contributions of treatment factors are noted for LOS. Sensitivity analyses identified no significant changes to the overall result.

## Discussion

This study presents a preliminary but comprehensive analysis of black/white racial disparities in treatment, mortality, and LOS for common general surgical operations. Fig. 3 provides a conceptual model of the contributors of these disparities based on the data available in the NIS. Disparities in delivery of surgery, time to surgery, LOS, and inpatient mortality were seen in all six diagnoses. Only the mortality disparities (except for appendicitis) and disparities in treatment and LOS for perforations were fully attributable to nonrace factors in the model.

The comparison of six general surgical diagnoses side by side allows for certain lessons to be gleaned that could guide interventions and further study. For example, the role of socioeconomic factors is particularly pronounced in the disparity in receipt of hernia surgery. The components of that factor group—income, insurance, weekend admission, and elective admission—are inherently more significant to hernia repairs (where many surgeries are elective and require insurance). This suggests a role for expanding insurance coverage in addressing disparities, which is supported by the literature in other diagnoses. 19 20 21 Second, the fact that there is no disparity in delivery of surgery for perforations after multivariable adjustment (although black patients do wait longer) suggests that it is possible to deliver surgery equitably. This, along with the observed role of hospital factors in explaining longer waits among black patients suggest that institutional-level quality improvement programs could be implemented to improve efficiency in getting patients to surgery.

The primary findings of this study—disparate delivery of surgery and longer wait times that appear both multifactorial and partially inexplicable—are consistent with the literature. Black patients are less likely to have surgery for a variety of indications 6 7 8 9 10 11 12 13 and wait longer even for fixation after hip fractures. 22 Occasionally, these disadvantages have been attributed wholly to individual covariates (such as socioeconomic status), 23 but comprehensive mediation analysis such as the one performed here support the multifactorial view of disparities that is gaining increasing prominence. 6 15 The observation that portions of the disparities are alternatively attributable to multiple categories of factors, supports a “Swiss cheese” model of the disparity, where multiple potential intervention points could lead to improvements. This should not come as a surprise; discrimination and disadvantage are multifaceted. 24

Disparities' multifactorial roots do not absolve physicians of responsibility for correcting them. 25 The only type of explanatory covariate that should fully reassure us about our performance would be valid medical contraindications to surgery—which would exclude poverty, insurance status, and many hospital factors. Even in the context of many comorbidities, appendicitis, 26 cholecystitis, 27 gallstone pancreatitis, 28 and perforations are indications for surgery. Nonoperative management is an option in select cases of any of the six diagnoses being evaluated, 29 30 but this does not justify disproportionate selection of minorities for nonoperative management. The delays in surgery seem even harder to rationalize, and delays have been associated with worse outcomes in cholecystitis, 31 appendicitis, 32 hernias, 33 perforations, 34 and obstructions. 35

Some of the disparities may appear to be small in magnitude, but perspective can be gained by considering the individual and societal impact of a single day's extension in hospital stay. To individuals with adequate health insurance coverage, paid sick leave, and effective options for childcare, an additional hospital stay is a manageable cost. To patients with unsteady employment where missing a day could put the job at risk, unavailable or unsafe childcare options, and high deductible insurance plans (if any), an additional day in the hospital can be extremely costly. Minority patients are more likely to face each of these vulnerabilities 36 37 38 and to the extent surgeons disproportionately add to their financial burdens, we may essentially contribute to national socioeconomic disparities.

Further descriptive studies could be beneficial to corroborate these findings and elucidate societal and patient-level costs of delays and nonoperative management. Institutional-level studies may also provide more detail about the causes or mechanisms underlying the observed disparities. The slow or negligible pace of improvement may be due to valid medical reasons for treatment differences or structural factors that institution-level studies could identify. Alternatively, deeply rooted societal challenges could be the proximal cause of the disparities—for example, if the difference in LOS is attributable to differences in homelessness and placement difficulties. Lastly, the possibility must be entertained that the treatment disparities observed here may be yet another example of racial discrimination. A more proactive way to address these questions would be to implement institutional quality improvement interventions to decrease wait times for inpatient surgery with an emphasis on disadvantaged patients. Publication of strategies and obstacles for such improvements would be a great step toward understanding and correcting the observed disparities.

### Limitations

This study has limitations. The NIS itself has severe limitations, to the point that this study should be seen as a hypothesis-generating study rather than definitive proof of cause and effect. The NIS is an administrative database, and as such relies on data designed primarily for billing assessments rather than detailed medical analyses. An attempt was made to include all relevant variables from the NIS. However, no account could be made of patients not entered into the database, potential inaccuracies in the source data, repeated admissions of the same patient, or critical patient-level factors such as education level (although previous studies have found a negligible role of patient refusal in cancer surgery disparities). 6 More definitive assessments of underlying causes of these disparities could be done within institutions, including root cause analysis. This is especially true given the single analytical format of this study across six diagnoses; validated diagnosis-specific details would be available at the institutional level and could guide different analytical strategies. For example, with some diagnoses there is evidence that more precise estimates of socioeconomic status (such as census-block-level social deprivation indices, or individual income data) would carry more explanatory value in modeling. 39 Although the mediation analysis was performed on only years 2002 to 2011, the following factors require consideration when interpreting the trends over time: the changes in the NIS sampling in 2012, the transition from ICD-9 to ICD-10 in the NIS in 2015, and the overall changes in general surgery as a field in the last 30 years. 40 There is conceptual overlap among some of the variables—particularly between type of surgery performed and presenting medical factors—so the contributions of these individual variables should be expected to overlap as well. Lastly, for the mediation analysis, although confidence intervals and *p* -values can be calculated through empirical estimations of variance (such as bootstrapping), 19 no testing was performed in this case. The mediation analysis must therefore be interpreted as a conceptual approximation rather than a precise assessment of contributions.

## Conclusion

Race/ethnicity-related treatment disparities are present in patients with six common indications for general surgical procedures (appendicitis, cholecystitis, gallstone pancreatitis, hernias, obstructions, and perforations). These disparities are only partially attributable to differences in clinical context of presentation and hospital factors. Treatment disparities in some cases may contribute to LOS disparities and pronounced disparities in delivery of surgery are present. Surgeons should plan institution-level studies and interventions to definitively measure, explain, and potentially correct treatment disparities.
