# Supplementary material for: Inefficiency in Delivery of General Surgery to Black Patients: A National Inpatient Sample Study
Source: Surg J (N Y). 2023 Dec 19;9(4):e123–34. doi: 10.1055/s-0043-1777811 (PMC10730284; doi:10.1055/s-0043-1777811)
Supplement: Supplementary file 1 — Supplementary Material [file 10-1055-s-0043-1777811-s2300021.pdf]

Supplementary Appendix 1 Diagnosis and Procedure Codes: ICD-9 (All Years Prior to 2016)

		Summary or general pattern*	ICD9 code short description**	ICD9 code in NIS	Mortality
Appendicitis	Lowest mortality	Acute appendicitis NOS, other appendicitis	Other appendicitis, Acute appendicitis NOS	542, 5409	0.05%
	Intermediate mortality	Appendicitis NOS	Appendicitis NOS	541	0.22%
	Highest mortality	Perforated appendicitis	Ac append w peritonitis, Abscess of appendix	5400, 5401	0.49%
	Least invasive or most definitive surgery	Laparoscopy	Appendectomy NOS, Lap appendectomy, Lap incid appendectomy	4701, 470, 471, 4711, 0470***	
	Temporizing surgery	Percutaneous Drainage	Drain appendiceal absc, Percu abdominal drainage	472, 5491	
	Difficult surgery - 1	Open, additional lysis of adhesions	Other appendectomy, Other incid appendectomy, Lap periton adhesiolysis, Oth periton adhesiolysis	4709, 5451, 5452, 545, 0545, 4719, 5459	
	Difficult surgery - 2	Extra anatomic step - bowel resections	Laparoscopic cecectomy, Lap right hemicolectomy, Lap pt ex lrg intest NEC, Open cecectomy NEC, Opn rt hemicolectomy NEC, Percu abdominal drainage (in addition to surgery)	4572, 4573, 1739, 1733, 1732, 5491	
Cholecystitis	Lowest mortality	Cholecystitis, no obstruction mentioned	Cholelith w ac cholecyst, Cholelith w cholecys NEC, Gal&bil cal w/oth w/o ob, Gal&bil cal w/ac&chr w/o, Chronic cholecystitis	57400, 57410, 57470, 57480, 57511	0.48%
	Intermediate mortality	Cholecystitis, with ductal involvement, no obstruction	Choledocholith/ac gb inf, Choledochlith/gb inf NEC, Gall&bil cal w/ac w/o ob, Cholecystitis NOS	57430, 57440, 57460, 57510	0.84%
	Highest mortality	Cholecystitis with obstruction mentioned	Acute cholecystitis, Obstruction gallbladder, Hydrops of gallbladder, Cholelith/ac gb inf-obst, Cholelith/gb inf NEC-obs, Choledochlith/ac gb-obst, Choledochlith/gb NEC-obs, Gall&bil cal w/ac w obs, Gall&bil cal w/oth w obs, Gal&bil cal w/ac&ch w ob, Acte & chr cholecystitis	5750, 5752, 5753, 57401, 57411, 57431, 57441, 57461, 57471, 57481, 57512	1.14%
	Minimally invasive or less invasive surgery	Definite Laparoscopy	Laparoscopic cholecystec	5123	
	Temporizing surgery	Percutaneous Drainage	Percutan aspiration gb, Trocar cholecystostomy, Cholecystostomy NEC, Cholecystotomy NEC	5101, 5102, 5103, 5104	
	Difficult surgery - 1	Open/partial cholecystectomy	Oth part cholecystectomy, Cholecystectomy, Lap part cholecystectomy	5122, 5124, 5121	
	Difficult surgery - 2	Surgery on the bile duct	Choledochoenterostomy, Hepatic duct-gi anastom, Bile duct anastomos NEC, Simple sut-common duct, Clos biliary fistul NEC	5137, 5136, 5171, 5139, 5193	

(*Continued*)

 © 2023. The Author(s).

Supplementary Appendix 1 (*Continued*)

		Summary or general pattern*	ICD9 code short description**	ICD9 code in NIS	Mortality
Gallstone Pancreatitis	Lowest mortality	Chronic pancreatitis with additional diagnosis of gallstones (non cholecystitis)	Chronic pancreatitis + Cholangitis, Obstruction of bile duct, Cholelithiasis NOS, Cholelithias NOS w obstr, Choledocholithiasis NOS, Choledochlith NOS w obst, Gall&bil cal w/o cho w/o, Gall&bil cal w/o ch w ob	5771 (primary) + 57420, 57421, 57450, 57451, 57490, 57491, 5761, 5762	0.83%
	Intermediate mortality	Any pancreatitis with additional diagnosis of cholecystitis	Acute pancreatitis, Chronic pancreatitis + Acute cholecystitis, Obstruction gallbladder, Hydrops of gallbladder, Cholelith w ac cholecyst, Cholelith/ac gb inf-obst, Cholelith w cholecys NEC, Cholelith/gb inf NEC-obs, Choledocholith/ac gb inf, Choledochlith/ac gb-obst, Choledochlith/gb inf NEC, Choledochlith/gb NEC-obs, Gall&bil cal w/ac w/o ob, Gall&bil cal w/ac w obs, Gal&bil cal w/oth w/o ob, Gall&bil cal w/oth w obs, Gal&bil cal w/ac&chr w/o, Gal&bil cal w/ac&ch w ob, Cholecystitis NOS, Chronic cholecystitis, Acte & chr cholecystitis, Gastrotomy	5770, 5771 (primary) + 57400, 57401, 57410, 57411, 57430, 57431, 57440, 57441, 57460, 57461, 57470, 57471, 57480, 57481, 5750, 57510, 57511, 57512, 5752, 5753	1.16%
	Highest mortality	Acute pancreatitis with additional diagnosis of gallstones (non cholecystitis)	Acute pancreatitis + Cholangitis, Obstruction of bile duct, Cholelithiasis NOS, Cholelithias NOS w obstr, Choledocholithiasis NOS, Choledochlith NOS w obst, Gall&bil cal w/o cho w/o, Gall&bil cal w/o ch w ob	5770 (primary) + 57420, 57421, 57450, 57451, 57490, 57491, 5761, 5762	1.36%
	Least invasive or most definitive surgery	Laparoscopy or open cholecystectomy	Oth part cholecystectomy, Cholecystectomy, Laparoscopic cholecystec, Lap part cholecystectomy	5122, 5124, 5121, 5123	
	Temporizing surgery	ERCP or percutaneous cholangiogram	Endosc retro cholangiopa, Endosc retro cholangio, Sphincter of oddi dilat, Pancreat sphincterotom, Pancreat sphincteroplas, Endosc dilation ampulla, Endosc sphincterotomy, Endosc inser stent bile, Endosc remove bile stone, Endosc retro pancreatog, Perc hepat Cholangiogram	5213, 5110, 5184, 5183, 5111, 5182, 5181, 5185, 5188, 5187, 8751	
	Difficult surgery - 1	Surgery to bile duct	Choledochoenterostomy, Hepatic duct-gi anastom, Bile duct anastomos NEC, CDE for calculus remov, CDE for obstruction NEC, Choledochohepat intubat, Incis obstr bile duc NEC, Common duct exploration, Bile duct incision NEC	5136, 5137, 5139, 5141, 5142, 5143, 5149, 5151, 5159	

Supplementary Appendix 1 (*Continued*)

		Summary or general pattern*	ICD9 code short description**	ICD9 code in NIS	Mortality
	Difficult surgery - 2	Pancreatic surgery or other laparotomy	Pancreat cyst marsupiali, Int drain pancreat cyst, Total pancreatectomy, Rad pancreaticoduodenect, Proximal pancreatectomy, Distal pancreatectomy, Rad subtot pancreatectom, Partial pancreatect NEC, Exploratory laparotomy	523, 524, 5251, 5252, 5253, 5259, 526, 527, 5411	
Obstructions	Lowest mortality	Intussusception	Intussusception	5600	0.90%
	Intermediate mortality	All others	Duodenal obstruction NEC, Intestinal obstruct NOS, Impaction intestine NOS, Intestinal adhes w obstr, Intestinal obstruct NEC	5373, 5609, 56030, 56081, 56089	2.61%
	Highest mortality	Volvulus	Volvulus of intestine	5602	5.85%
	Least invasive or most definitive surgery	Exploration/ lysis of adhesions / hernia repairs	Large bowel fixation NEC, Intra-ab bowel manip NOS, Intra-abd sm bowel manip, Intra-abd lg bowel manip, Other appendectomy, Laparoscopic cholecystec, Open incis hern-grft NEC, Other hernia repair, Exploratory laparotomy, Laparotomy NEC, Laparoscopy, Destruct peritoneal tiss, Lap periton adhesiolysis, Oth periton adhesiolysis	5459, 545, 5451, 4681, 5411, 4682, 5421, 544, 5123, 539, 4664, 4709, 4680, 5419, 5361	
	Temporizing surgery	Endoscopy, percutaneous drainage/diversion	Other gastrostomy, Clos small bowel biopsy, Endosc lg bowel thru st, Clos large bowel biopsy, Endo polpectomy lrge int, Dilation of intestine, Endo insrt colonic stent, Closed rectal biopsy, Percu abdominal drainage, Insert gastric tube NEC	9607, 4685, 4525, 5491, 4542, 4311, 4319, 4824, 4522, 4686, 4514	
	Difficult surgery - 1	Simple bowel resections/repair	Small bowel incision NEC, Lg bowel exteriorization, Small bowel suture NEC, Suture lg bowel lacerat, Repair of intestine NEC	4673, 4603, 4679, 4502, 4675	
	Difficult surgery - 2	Exploratory laparotomies, additional surgical steps	Lap sigmoidectomy, Gastroenterostomy NEC, Mult seg sm bowel excis, Part sm bowel resect NEC, Open cecectomy NEC, Opn rt hemicolectomy NEC, Opn transv colon res NEC, Opn lft hemicolectmy NEC, Open sigmoidectomy NEC, Prt lg intes exc NEC/NOS, Sm-to-sm bowel anastom, Small-to-large bowel NEC, Sm bowel exteriorization, Colostomy NOS, Temporary colostomy, Permanent colostomy	4562, 4573, 4576, 4572, 4575, 4561, 4591, 4579, 4593, 4610, 4439, 4601, 4613, 1736, 4611, 4574	

(*Continued*)

 © 2023. The Author(s).

The Surgery Journal © 2023. The Author(s).

Supplementary Appendix 1 (*Continued*)

		Summary or general pattern*	ICD9 code short description**	ICD9 code in NIS	Mortality
Abdominal Wall Hernias	Lowest mortality	No obstruction or gangrene	Umbilical hernia, Hernia NEC, Hernia NOS, Unilat femoral hernia, Recur unil femoral hern, Bilateral femoral hernia, Recur bilat femoral hern, Ventral hernia NOS, Incisional hernia, Unilat inguinal hernia, Recur unilat inguin hern, Bilat inguinal hernia, Recur bilat inguin hern,	5531, 5538, 5539, 55090, 55091, 55092, 55093, 55300, 55301, 55302, 55303, 55320, 55321	0.35%
	Intermediate mortality	Unqualified, may have obstruction	Umbilical hernia w obstr, Hernia, site NEC w obstr, Hernia, site NOS w obstr, Rec unil fem hern w obst, Bil femoral hern w obstr, Rec bil fem hern w obstr, Obstr ventral hernia NOS, Obstr incisional hernia, Unilat ing hernia w obst, Recur unil ing hern-obst, Bilat ing hernia w obst, Recur bil ing hern-obstr, Unil femoral hern w obst,	5521, 5528, 5529, 55010, 55011, 55012, 55013, 55200, 55201, 55202, 55203, 55220, 55221	1.73%
	Highest mortality	Perforation or abscess,	Umbilical hernia w gangr, Hernia, site NEC w gangr, Hernia, site NOS w gangr, Unilat ing hernia w gang, Recur unil ing hern-gang, Bilat ing hernia w gang, Recur bil ing hern-gang, Unil femoral hern w gang, Rec unil fem hern w gang, Bilat fem hern w gang, Recur bil fem hern-gang, Gangr ventral hernia NOS, Gangr incisional hernia	5511, 5518, 5519, 55000, 55001, 55002, 55003, 55100, 55101, 55102, 55103, 55120, 55121	7.73%
	Least invasive or most definitive surgery	Mesh used	Lap indir ing hern-graft, Lap ing hern-graft NOS, Lap bil dir ing hrn-grft, Lap bi dr/ind ing hrn-gr, Lap bil ing hern-grf NOS, Opn dir ing hern-gft NEC, Opn ind ing hern-gft NEC, Ing hernia rep-graft NOS, Opn bi dr ing hrn-gr NEC, Op bi in ing hrn-grf NEC, Op bi dr/in ig hr-gr NEC, Bil ing hrn rep-grft NOS, Unil femor hrn rep-grft, Bil fem hern repair-grft, Opn rep umb hrn-grft NEC, Lap umbil hernia-graft, Open incis hern-grft NEC, Lap incis hern repr-grft, Lap hern ant abd-gft NEC, Opn hern ant abd-grf NEC, Lap dir ing hern-graft, Lap bi indir ing hrn-grf,	5361, 5369, 5304, 5305, 5303, 5341, 5362, 5321, 5314, 5363, 5317, 5316, 5315, 5342, 1712, 1713, 1724, 1723, 1721, 5331, 1711, 1722	

Supplementary Appendix 1 (*Continued*)

		Summary or general pattern*	ICD9 code short description**	ICD9 code in NIS	Mortality
	Temporizing surgery	No mesh used	Unilat ing hern rep NOS, Opn rep dir ing hern NEC, Opn rep ind ing hern NEC, Bilat ing hernia rep NOS, Opn bil dir ing hern NEC, Opn bil ind ing hern NEC, Opn bi dr/in ing hrn NEC, Unil femor hern rep NEC, Lap umbilical hernia NEC, Open rep umbil hern NEC, Pericolost hernia repair, Incisional hernia repair, Abd wall hern repair NEC, Other hernia repair, Abdomen wall repair NEC	537, 5351, 5349, 5359, 5302, 5301, 5300, 5329, 539, 5311, 5310, 5313, 5312, 5384, 5343, 4642, 5472	
	Difficult surgery - 1	Additional exploratory procedures; lysis of adhesions, intestinal repairs, minor resections	Gastric repair NEC, Intraop gastric manipul, Small bowel incision NEC, Opn rt hemicolectomy NEC, Sm bowel stoma revision, Small bowel suture NEC, Suture lg bowel lacerat, Repair of intestine NEC, Intra-abd sm bowel manip, Intra-abd lg bowel manip, Lap appendectomy, cholecystec, Abdominal wall incision, Exploratory laparotomy, Laparoscopy, Destruct peritoneal tiss, Lap periton adhesiolysis, Oth periton adhesiolysis, Other appendectomy	545, 5459, 5451, 4673, 4573, 4681, 4679, 4675, 544, 5411, 5421, 540, 4701, 4469, 4492, 4502, 4682, 5122, 4641, 4709	
	Difficult surgery - 2	Resection performed	Opn/oth part gastrectomy, Local excis sm bowel NEC, Mult seg sm bowel excis, Part sm bowel resect NEC, Open cecectomy NEC, Opn transv colon res NEC, Opn lft hemicolectmy NEC, Open sigmoidectomy NEC, Prt lg intes exc NEC/NOS, Sm-to-sm bowel anastom, Resect ext seg sm bowel,	4562, 4576, 4561, 4579, 4572, 4574, 4591, 4575, 4533, 4389, 4602	
Perforations	Lowest mortality	Gastroduodenal or marginal	Ac stom ulc w perf-obst, Chr stomach ulcer w perf, Chr stom ulc w perf-obst, Ac duodenal ulcer w perf, Ac duoden ulc perf-obstr, Chr duoden ulcer w perf, Chr duoden ulc perf-obst, Ac peptic ulcer w perfor, Ac peptic ulc w perf-obs, Chr peptic ulcer w perf, Chr peptic ulc perf-obst, Ac marginal ulcer w perf, Ac margin ulc w perf-obs, Chr marginal ulc w perf, Chr margin ulc perf-obst	53251, 53111, 53150, 53151, 53210, 53211, 53250, 53251, 53310, 53311, 53350, 53351, 53410, 53411, 53450, 53451	8.98%
	Intermediate mortality	Gastroduodenal or marginal, generally also with hemorrhage	Ac stom ulc hem/perf-obs, Chr stomach ulc hem/perf, Chr stom ulc hem/perf-ob, Ac duoden ulc w hem/perf, Ac duod ulc hem/perf-obs, Chr duoden ulc hem/perf, Chr duod ulc hem/perf-ob, Ac peptic ulc w hem/perf, Ac pept ulc hem/perf-obs, Chr pept ulc w hem/perf, Chr pept ulc hem/perf-ob, Ac margin ulc w hem/perf, Ac marg ulc hem/perf-obs, Chr margin ulc hem/perf, Chr marg ulc hem/perf-ob	53261, 53121, 53160, 53161, 53220, 53221, 53260, 53261, 53320, 53321, 53360, 53361, 53420, 53421, 53460, 53461	17.92%

(*Continued*)

© 2023. The Author(s).

Supplementary Appendix 1 (*Continued*)

		Summary or general pattern*	ICD9 code short description**	ICD9 code in NIS	Mortality
	Highest mortality	Intestinal	Perforation of intestine	56983	19.08%
	Least invasive or most definitive surgery	Laparotomy or laparoscopy, repairs, acid reducing procedures	Vagotomy NOS, Truncal vagotomy, Highly select vagotomy, Selective vagotomy NEC, Open gastric biopsy, Other pyloroplasty, Suture peptic ulcer NOS, Sut gastric ulcer site, Suture duoden ulcer site, Suture gastric lacerat, Gastric repair NEC, Duodenal incision, Gastrotomy, Small bowel incision NEC, Large bowel incision, Open small bowel biopsy, Small bowel fixation NEC, Duodenal lacerat suture, Small bowel suture NEC, Suture lg bowel lacerat, Repair of intestine NEC, Exploratory laparotomy, Reopen recent lap site, Laparotomy NEC, Laparoscopy, Lap periton adhesiolysis, Omental repair NEC	4442, 4441, 4429, 4469, 4679, 4401, 4673, 5411, 4675, 4461, 4400, 5474, 4440, 458, 4415, 5419, 4662, 5421, 4403, 4671, 4402, 5451, 430, 4501, 4502, 4503, 4515, 5412, 4672, 433, 4499	
	Temporizing surgery	Drainage, diversion, dilation, or other endoscopy	Other gastrostomy, Closed gastric biopsy, Endosc control gast hem, Egd with closed biopsy, Percu abdominal drainage, Insert gastric tube NEC	4516, 4443, 5491, 9607, 4414, 4319, 4311	
	Difficult surgery - 1	Partial resections, ostomies	Lap right hemicolectomy, Lap sigmoidectomy, Proximal gastrectomy, Distal gastrectomy, Part gastrec w jej anast, Revision gastric anastom, Local gastr excision NEC, Opn/oth part gastrectomy, Gastroenterostomy NEC, Laparoscop gastroplasty, Oth excise duodenum les, Part sm bowel resect NEC, Open cecectomy NEC, Opn rt hemicolectomy NEC, Opn transv colon res NEC, Opn lft hemicolectmy NEC, Open sigmoidectomy NEC, Prt lg intes exc NEC/NOS, Sm-to-sm bowel anastom, Small-to-large bowel NEC, Sm bowel exteriorization, Resect ext seg sm bowel, Lg bowel exteriorization, Colostomy NOS, Temporary colostomy, Permanent colostomy, Ileostomy NOS, Enterostomy NEC, Ant rect resect w colost, Anterior rect resect NEC, Rectal resection NEC	4576, 4562, 437, 4573, 4575, 4342, 436, 4579, 4389, 4572, 4574, 4439, 4610, 4603, 445, 4531, 4611, 4601, 4613, 435, 4869, 4862, 4620, 1736, 1733, 4591, 4593, 4639, 4468, 4863, 4602	
	Difficult surgery - 2	Total resections of stomach, colon or small bowel	Total gastrectomy NEC, Total removal sm bowel, Op tot intr-abd colectmy	4582, 4399, 4563	

*Mortality groups were developed empirically based on observed mortality by diagnosis; the grouped codes do not always fall under one concept, but a general heading is provided. Surgical procedure codes were examined by diagnosis and those that were performed on more than 1/1000 of patients with the diagnosis were grouped into general conceptual headings given above. Subjects with more than one procedure were classified according to the most invasive procedure performed.

**"ICD-9-CM Diagnosis and Procedure Codes: Abbreviated and Full Code Titles." Centers for Medicare and Medicaid Services. Accessed 12/12/22. URL: https://www.cms.gov/medicare/coding-billing/icd-10-codes/icd-9-cm-diagnosis-procedure-codes-abbreviated-and-full-code-titles.

***In some cases alternative forms of the same codes appeared in NIS (such as codes starting with 47 for appendectomy; in these cases the codes were confirmed against the diagnosis listed for the NIS subject.

 © 2023. The Author(s).

Diagnosis and Procedure Codes: ICD-10 (2016–2017 Only)

		Summary or general heading*	Shortened ICD 10 Text**	ICD10 codes	Mortality
Appendicitis	Lowest mortality	Unspecified acute, Other	Unspecified acute appendicitis; Other acute appendicitis; Other appendicitis;	K3580, K3589, K36	0.07%
	Intermediate mortality	Unqualified	Acute appendicitis with localized peritonitis; Unspecified appendicitis;	K37, K353	0.16%
	Highest mortality	Perforated/ abscess	Acute appendicitis with generalized peritonitis;	K352	0.27%
	Least invasive or most definitive surgery	Laparoscopy or other MIS appendectomy	Excision of Appendix, Percutaneous Endoscopic Approach, Diagnostic; Excision of Appendix, Percutaneous Endoscopic Approach; Resection of Appendix, Percutaneous Endoscopic Approach; Resection of Appendix, Via Natural or Artificial Opening Endoscopic;	0DTJ4ZZ, 0DTJ8ZZ, 0DBJ4ZZ, 0DBJ4ZX	
	Temporizing surgery	Percutaneous Drainage	Drainage of Appendix, Percutaneous Approach, Diagnostic; Drainage of Appendix, Percutaneous Approach; Drainage of Appendix with Drainage Device, Percutaneous Endoscopic Approach; Drainage of Appendix, Percutaneous Endoscopic Approach; Drainage of Peritoneum with Drainage Device, Percutaneous Approach; Excision of Appendix, Percutaneous Endoscopic Approach; Drainage of Pelvic Region Subcutaneous Tissue and Fascia with Drainage Device, Percutaneous Approach; Drainage of Abdominal Wall, Percutaneous Approach; Drainage of Peritoneal Cavity with Drainage Device, Open Approach; Drainage of Peritoneal Cavity, Percutaneous Approach, Diagnostic; Drainage of Peritoneal Cavity, Percutaneous Approach; Drainage of Peritoneal Cavity with Drainage Device, Percutaneous Endoscopic Approach; Drainage of Peritoneal Cavity, Percutaneous Endoscopic Approach; Drainage of Retroperitoneum with Drainage Device, Percutaneous Approach; Drainage of Pelvic Cavity, Percutaneous Approach; Drainage of Pelvic Cavity with Drainage Device, Percutaneous Endoscopic Approach;	0W9G3ZZ, 0D9W30Z, 0W9G40Z, 0D9J3ZZ, 0DBJ4ZZ, 0D9J4ZZ, 0W9G3ZX, 0D9J40Z, 0W9J3ZZ, 0W9H30Z, 0W9J40Z, 0D9J3ZX, 0W9F3ZZ, 0W9G00Z, 0W9G4ZZ, 0J9C30Z	
	Difficult surgery - 1	Open, or cecum taken	Excision of Cecum, Open Approach; Excision of Cecum, Percutaneous Endoscopic Approach; Excision of Appendix, Open Approach; Resection of Cecum, Open Approach; Resection of Appendix, Open Approach; Resection of Cecum, Percutaneous Endoscopic Approach;	0DTJ0ZZ, 0DTH0ZZ, 0DBH4ZZ, 0DBH0ZZ, 0DBJ0ZZ, 0DTH4ZZ,	
	Difficult surgery - 2	Additional anatomic steps/biopsies	Excision of Stomach, Via Natural or Artificial Opening Endoscopic, Diagnostic; Excision of Small Intestine, Open Approach; Excision of Right Large Intestine, Open Approach; Excision of Ascending Colon, Open Approach; Resection of Right Large Intestine, Open Approach; Resection of Right Large Intestine, Percutaneous Endoscopic Approach;	0DTF0ZZ, 0DTF4ZZ, 0DBF0ZZ, 0DB80ZZ, 0DBK0ZZ, 0DB68ZX	
Cholecystitis	Lowest mortality	No obvious pattern	Calculus of gallbladder w acute cholecyst w/o obstruction; Calculus of gallbladder w acute cholecystitis w obstruction; Calculus of gallbladder w chronic cholecyst w/o obstruction; Calculus of gallbladder w chronic cholecyst w obstruction; Calculus of GB w acute and chronic cholecyst w/o obstruction; Calculus of gallbladder w oth cholecystitis with obstruction; Calculus of bile duct w cholecystitis, unsp, w obstruction; Calculus of bile duct w acute cholecystitis w/o obstruction; Calculus of bile duct w chronic cholecyst w/o obstruction; Calculus of bile duct w acute and chronic cholecyst w obst; Calculus of GB and bile duct w acute cholecyst w/o obst; Calculus of GB and bile	K8019, K820, K829, K8064, K8066, K828, K8010, K8062, K8000, K811, K8044, K8041, K8042, K8047, K8001, K8012, K8011, K8067	0.33%

(*Continued*)

(*Continued*)

		Summary or general heading*	Shortened ICD 10 Text**	ICD10 codes	Mortality
			duct w chronic cholecyst w/o obst; Calculus of GB and bile duct w ac and chr cholecyst w/o obst; Calculus of GB and bile duct w ac and chr cholecyst w obst; Chronic cholecystitis; Obstruction of gallbladder; Other specified diseases of gallbladder; Disease of gallbladder, unspecified;		
	Intermediate mortality	No obvious pattern	Calculus of GB w acute and chronic cholecyst w obstruction; Calculus of gallbladder w oth cholecystitis w/o obstruction; Calculus of bile duct w cholecystitis, unsp, w/o obstruction; Calculus of bile duct w acute cholecystitis with obstruction; Calculus of bile duct w chronic cholecystitis w obstruction; Calculus of bile duct w acute and chronic cholecyst w/o obst; Calculus of GB and bile duct w cholecyst, unsp, w/o obst; Calculus of GB and bile duct w cholecyst, unsp, w obst; Calculus of GB and bile duct w acute cholecyst w obstruction; Calculus of GB and bile duct w chronic cholecyst w obst; Acute cholecystitis; Acute cholecystitis with chronic cholecystitis; Cholecystitis, unspecified; Hydrops of gallbladder;	K812, K8043, K8060, K8063, K8013, K8040, K810, K8046, K8061, K819, K8065, K8018, K8045, K821	0.68%
	Highest mortality	No obvious pattern	Other cholelithiasis with obstruction; Perforation of gallbladder; Fistula of gallbladder; Cholesterolosis of gallbladder;	K824, K822, K8081, K823	2.22%
	Least invasive or most definitive surgery	Laparoscopy	Excision of Gallbladder, Percutaneous Endoscopic Approach, Diagnostic; Excision of Gallbladder, Percutaneous Endoscopic Approach; Resection of Gallbladder, Percutaneous Endoscopic Approach;	0FT44ZZ, 0FB44ZZ, 0FB44ZX	
	Temporizing surgery	Percutaneous Drainage	Drainage of Gallbladder with Drainage Device, Percutaneous Approach; Drainage of Gallbladder, Percutaneous Approach, Diagnostic; Drainage of Gallbladder, Percutaneous Approach;	0F9430Z, 5A1D60Z, 0F943ZZ, 0F943ZX	
	Difficult surgery - 1	Open, endoscopic CBD biopsy	Drainage of Gallbladder with Drainage Device, Open Approach; Excision of Gallbladder, Open Approach, Diagnostic; Excision of Gallbladder, Open Approach; Excision of Common Bile Duct, Via Natural or Artificial Opening Endoscopic, Diagnostic; Resection of Gallbladder, Open Approach;	0FT40ZZ, 0FB40ZZ, 0F9400Z, 0FB98ZX, 0FB40ZX	
	Difficult surgery - 2	Additional anatomic steps	Drainage of Stomach with Drainage Device, Via Natural or Artificial Opening; Excision of Esophagus, Via Natural or Artificial Opening Endoscopic, Diagnostic; Excision of Stomach, Via Natural or Artificial Opening Endoscopic, Diagnostic; Excision of Stomach, Pylorus, Via Natural or Artificial Opening Endoscopic, Diagnostic; Excision of Duodenum, Via Natural or Artificial Opening Endoscopic, Diagnostic; Excision of Large Intestine, Via Natural or Artificial Opening Endoscopic, Diagnostic; Bypass Common Bile Duct to Duodenum, Open Approach; Bypass Common Bile Duct to Small Intestine, Open Approach; Excision of Liver, Open Approach; Repair Common Bile Duct, Open Approach;	0DB68ZX, 0DB98ZX, 0DB78ZX, 0DB58ZX, 0FB00ZZ, 0F190ZB, 0FQ90ZZ, 0F190Z3, 0D9670Z, 0DBE8ZX	
Gallstone Pancreatitis	Lowest mortality	No necrosis	Biliary acute pancreatitis; Biliary acute pancreatitis without necrosis or infection; Acute pancreatitis, unspecified; Acute pancreatitis without necrosis or infection, unsp;	K851, K8510, K859, K8590	0.75%
	Intermediate mortality	Others	Biliary acute pancreatitis with uninfected necrosis; Acute pancreatitis with uninfected necrosis, unspecified; Acute pancreatitis with infected necrosis, unspecified. Any additional code consistent with biliary lithiasis required to include unspecified pancreatitis	K8591, K8511, K8592	3.36%

(*Continued*)

		Summary or general heading*	Shortened ICD 10 Text**	ICD10 codes	Mortality
	Highest mortality	Biliary with infected necrosis	Biliary acute pancreatitis with infected necrosis;	K8512	12.77%
	Least invasive or most definitive surgery	Laparoscopy or open chole	Excision of Gallbladder, Percutaneous Endoscopic Approach; Resection of Gallbladder, Open Approach; Resection of Gallbladder, Percutaneous Endoscopic Approach;	0FT44ZZ, 0FT40ZZ, 0FB44ZZ	
	Temporizing surgery	ERCP	Dilation of Common Bile Duct with Intraluminal Device, Via Natural or Artificial Opening Endoscopic; Dilation of Common Bile Duct, Via Natural or Artificial Opening Endoscopic; Dilation of Ampulla of Vater, Via Natural or Artificial Opening Endoscopic; Dilation of Pancreatic Duct with Intraluminal Device, Via Natural or Artificial Opening Endoscopic; Dilation of Pancreatic Duct, Via Natural or Artificial Opening Endoscopic; Drainage of Common Bile Duct, Via Natural or Artificial Opening Endoscopic; Drainage of Ampulla of Vater, Via Natural or Artificial Opening Endoscopic; Drainage of Pancreas with Drainage Device, Percutaneous Approach; Drainage of Pancreas, Percutaneous Endoscopic Approach; Excision of Pancreas, Percutaneous Endoscopic Approach; Extirpation of Matter from Common Bile Duct, Via Natural or Artificial Opening Endoscopic; Extirpation of Matter from Ampulla of Vater, Via Natural or Artificial Opening Endoscopic; Extirpation of Matter from Pancreatic Duct, Via Natural or Artificial Opening Endoscopic; Inspection of Hepatobiliary Duct, Via Natural or Artificial Opening Endoscopic; Inspection of Pancreatic Duct, Via Natural or Artificial Opening Endoscopic; Removal of Intraluminal Device from Pancreatic Duct, Via Natural or Artificial Opening Endoscopic;	0FC98ZZ, 0F798ZZ, 0F798DZ, 0FJB8ZZ, 0F7D8DZ, 0FJD8ZZ, 0F9C8ZZ, 0F7C8ZZ, 0FCD8ZZ, 0F7D8ZZ, 0F998ZZ, 0FPD8DZ, 0F9G30Z, 0FCC8ZZ, 0F9G4ZZ, 0FBG4ZZ	
	Difficult surgery - 1	Surgery on bile duct, stomach	Excision of Stomach, Pylorus, Via Natural or Artificial Opening Endoscopic, Diagnostic; Excision of Common Bile Duct, Via Natural or Artificial Opening Endoscopic, Diagnostic;	0FB98ZX, 0DB78ZX	
	Difficult surgery - 2	Pancreatic surgery or drainage	Excision of Ampulla of Vater, Via Natural or Artificial Opening Endoscopic, Diagnostic; Excision of Pancreas, Open Approach; Drainage of Peritoneal Cavity with Drainage Device, Percutaneous Approach; Drainage of Peritoneal Cavity, Percutaneous Approach, Diagnostic; Drainage of Peritoneal Cavity, Percutaneous Approach;	0FBG0ZZ, 0W9G3ZZ, 0W9G3ZX, 0W9G30Z, 0FBC8ZX	
Abdominal Wall Hernias	Lowest mortality	No obvious pattern	Bi inguinal hernia, w gangrene, not specified as recurrent; Bi inguinal hernia, w/o obst or gangrene, not spcf as recur; Bilateral inguinal hernia, w/o obst or gangrene, recurrent; Unil inguinal hernia, w/o obst or gangr, not spcf as recur; Unilateral inguinal hernia, w/o obst or gangrene, recurrent; Bi femoral hernia, w obst, w/o gangrene, not spcf as recur; Bi femoral hernia, w/o obst or gangrene, not spcf as recur; Unilateral femoral hernia, w obst, w/o gangrene, recurrent; Unilateral femoral hernia, w/o obst or gangrene, recurrent; Umbilical hernia with obstruction, without gangrene; Umbilical hernia without obstruction or gangrene; Incisional hernia with obstruction, without gangrene; Incisional hernia without obstruction or gangrene; Parastomal hernia without obstruction or gangrene; Ventral hernia without obstruction or gangrene; Oth abdominal hernia without obstruction or gangrene; Unspecified abdominal hernia without obstruction or gangrene;	K430, K420, K4091, K439, K4090, K432, K4020, K429, K435, K4010, K4021, K4100, K4120, K4131, K4191, K458, K469	0.66%

(*Continued*)

 © 2023. The Author(s).

(*Continued*)

		Summary or general heading*	Shortened ICD 10 Text**	ICD10 codes	Mortality
	Intermediate mortality	No obvious pattern	Bi inguinal hernia, w obst, w/o gangrene, not spcf as recur; Bilateral inguinal hernia, w obst, w/o gangrene, recurrent; Unil inguinal hernia, w obst, w/o gangr, not spcf as recur; Unilateral inguinal hernia, w obst, w/o gangrene, recurrent; Unil inguinal hernia, w gangrene, not specified as recurrent; Unil femoral hernia, w obst, w/o gangrene, not spcf as recur; Unil femoral hernia, w/o obst or gangrene, not spcf as recur; Umbilical hernia with gangrene; Incisional hernia with gangrene; Parastomal hernia with obstruction, without gangrene; Other and unsp ventral hernia with obstruction, w/o gangrene; Other and unspecified ventral hernia with gangrene; without gangrene; Oth abdominal hernia with obstruction, without gangrene; Other specified abdominal hernia with gangrene; Unsp abdominal hernia with obstruction, without gangrene;	K431, K4040, K421, K437, K450, K4130, K4000, K451, K433, K460, K4031, K4030, K4001, K4190, K436	1.81%
Highest mortality		Gangrene present	Bilateral inguinal hernia, with gangrene, recurrent; Unilateral inguinal hernia, with gangrene, recurrent; Unil femoral hernia, w gangrene, not specified as recurrent; Unilateral femoral hernia, with gangrene, recurrent; Parastomal hernia with gangrene; Unspecified abdominal hernia with gangrene;	K4011, K4141, K434, K461, K4041, K4140	7.12%
Least invasive or most definitive surgery		Mesh used	Percutaneous Endoscopic Approach; Repair Esophagogastric Junction, Percutaneous Endoscopic Approach; Restriction of Esophagogastric Junction, Percutaneous Endoscopic Approach; Supplement Abdominal Wall with Synthetic Substitute, Open Approach; Supplement Abdominal Wall with Nonautologous Tissue Substitute, Open Approach; Supplement Abdominal Wall with Synthetic Substitute, Percutaneous Endoscopic Approach; Supplement Right Inguinal Region with Synthetic Substitute, Open Approach; Supplement Right Inguinal Region with Synthetic Substitute, Percutaneous Endoscopic Approach; Supplement Left Inguinal Region with Synthetic Substitute, Open Approach; Supplement Left Inguinal Region with Synthetic Substitute, Percutaneous Endoscopic Approach; Supplement Right Femoral Region with Synthetic Substitute, Open Approach; Supplement Left Femoral Region with Synthetic Substitute, Open Approach; Supplement Bilateral Inguinal Region with Synthetic Substitute, Open Approach; Supplement Bilateral Inguinal Region with Synthetic Substitute, Percutaneous Endoscopic Approach;	0WUF0JZ, 0WUF4JZ, 0YU50JZ, 0DV44ZZ, 0YU60JZ, 0YU54JZ, 0WUF0KZ, 0YUA4JZ, 0YU70JZ, 0YU64JZ, 0YUA0JZ, 0YU80JZ, 0DQ44ZZ	
Temporizing surgery		No mesh used	Repair Abdominal Wall, Open Approach; Repair Abdominal Wall, Percutaneous Endoscopic Approach; Repair Abdominal Wall, Stoma, External Approach; Repair Abdominal Wall, External Approach; Repair Right Inguinal Region, Open Approach; Repair Right Inguinal Region, Percutaneous Endoscopic Approach; Repair Left Inguinal Region, Open Approach; Repair Left Inguinal Region, Percutaneous Endoscopic Approach; Repair Right Femoral Region, Open Approach; Repair Left Femoral Region, Open Approach; Repair Bilateral Inguinal Region, Open Approach;	0WQF0ZZ, 0WQF4ZZ, 0YQ50ZZ, 0YQ60ZZ, 0YQ70ZZ, 0YQ80ZZ, 0YQ54ZZ, 0YQA0ZZ, 0YQ64ZZ, 0WQFXZ2, 0WQFXZZ,	
Difficult surgery - 1		No hernia repair	Bypass Stomach to Jejunum, Percutaneous Endoscopic Approach; Bypass Ileum to Cutaneous, Open Approach; Bypass Transverse Colon to Cutaneous, Open Approach; Drainage of Stomach with Drainage	0DN80ZZ, 0D9670Z, 0DNW0ZZ, 0DN84ZZ, 0DNW4ZZ, 0DQ80ZZ,	

 © 2023. The Author(s).

(*Continued*)

		Summary or general heading*	Shortened ICD 10 Text**	ICD10 codes	Mortality
			Device, Via Natural or Artificial Opening; Insertion of Feeding Device into Stomach, Via Natural or Artificial Opening; Inspection of Lower Intestinal Tract, Via Natural or Artificial Opening Endoscopic; Release Stomach, Open Approach; Release Stomach, Percutaneous Endoscopic Approach; Release Small Intestine, Open Approach; Release Small Intestine, Percutaneous Endoscopic Approach; Release Jejunum, Open Approach; Release Ileum, Open Approach; Release Ileum, Percutaneous Endoscopic Approach; Release Transverse Colon, Open Approach; Release Omentum, Open Approach; Release Peritoneum, Open Approach; Release Peritoneum, Percutaneous Endoscopic Approach; Repair Esophagogastric Junction, Open Approach; Repair Stomach, Percutaneous Endoscopic Approach; Repair Small Intestine, Open Approach; Repair Small Intestine, Percutaneous Endoscopic Approach; Repair Ileum, Open Approach; Reposition Stomach, Open Approach; Reposition Stomach, Percutaneous Endoscopic Approach; Reposition Ileum, Open Approach; Transfer Stomach to Esophagus, Percutaneous Endoscopic Approach; Release Right Abdomen Muscle, Open Approach; Release Left Abdomen Muscle, Open Approach; Drainage of Peritoneal Cavity, Percutaneous Approach, Diagnostic; Excision of Abdominal Wall, Open Approach; Inspection of Peritoneal Cavity, Percutaneous Endoscopic Approach; Inspection of Gastrointestinal Tract, Open Approach; Inspection of Gastrointestinal Tract, Percutaneous Endoscopic Approach;	0DS64ZZ, 0D1B0Z4, 0D164ZA, 0DJD8ZZ, 0DNB0ZZ, 0KNK0ZZ, 0DH67UZ, 0DS60ZZ, 0DX64Z5, 0DQB0ZZ, 0KNL0ZZ, 0WJP4ZZ, 0DQ64ZZ, 0WBF0ZZ, 0DN64ZZ, 0DNA0ZZ, 0WJP0ZZ, 0DQ40ZZ, 0DQ84ZZ, 0DSB0ZZ, 0DN60ZZ, 0W9G3ZX, 0WJG4ZZ, 0DNL0ZZ, 0D1L0Z4, 0DNU0ZZ, 0DNB4ZZ	
Difficult surgery - 2		Resection	Excision of Stomach, Open Approach; Excision of Stomach, Percutaneous Endoscopic Approach, Vertical; Excision of Small Intestine, Open Approach, Diagnostic; Excision of Small Intestine, Open Approach; Excision of Small Intestine, Percutaneous Endoscopic Approach; Excision of Duodenum, Via Natural or Artificial Opening Endoscopic, Diagnostic; Excision of Jejunum, Open Approach; Excision of Ileum, Open Approach; Excision of Large Intestine, Open Approach; Excision of Transverse Colon, Open Approach; Excision of Descending Colon, Open Approach; Excision of Sigmoid Colon, Open Approach; Release Large Intestine, Open Approach; Resection of Small Intestine, Open Approach; Resection of Ileum, Open Approach; Resection of Right Large Intestine, Open Approach; Resection of Cecum, Open Approach; Resection of Appendix, Open Approach; Resection of Appendix, Percutaneous Endoscopic Approach; Resection of Gallbladder, Percutaneous Endoscopic Approach; Removal of Synthetic Substitute from Abdominal Wall, Open Approach;	0DB80ZZ, 0DT80ZZ, 0DBB0ZZ, 0DTF0ZZ, 0DBA0ZZ, 0DNE0ZZ, 0DB84ZZ, 0DB64Z3, 0FT44ZZ, 0DTJ0ZZ, 0DBN0ZZ, 0DB80ZX, 0DBE0ZZ, 0DB98ZX, 0DBL0ZZ, 0DTH0ZZ, 0DBM0ZZ, 0DTB0ZZ, 0WPF0JZ, 0DB60ZZ, 0DTJ4ZZ	
Perforations	Lowest mortality	No obvious pattern	Acute gastric ulcer with perforation; Chronic or unspecified duodenal ulcer with perforation; Acute gastrojejunal ulcer with perforation; Chronic or unsp gastrojejunal ulcer w both hemor and perf; Dvtrcli of sm int w perforation and abscess w/o bleeding; Dvtrcli of sm int w perforation and abscess w bleeding;	K286, K251, K265, K281, K5700, K5701	2.94%

(*Continued*)

(*Continued*)

		Summary or general heading*	Shortened ICD 10 Text**	ICD10 codes	Mortality
	Intermediate mortality	No obvious pattern	Acute duodenal ulcer with perforation; Acute duodenal ulcer with both hemorrhage and perforation; Acute peptic ulcer, site unspecified, with perforation; Chronic or unsp peptic ulcer, site unsp, with perforation; Acute gastrojejunal ulcer w both hemorrhage and perforation; Chronic or unspecified gastrojejunal ulcer with perforation;	K271, K262, K282, K261, K275, K285	4.22%
	Highest mortality	No obvious pattern	Acute gastric ulcer with both hemorrhage and perforation; Chronic or unsp duodenal ulcer w both hemorrhage and perf; Chr or unsp peptic ulcer, site unsp, w both hemor and perf; Perforation of intestine (nontraumatic);	K252, K276, K266, K631	12.86%
	Least invasive surgery	No resection or stayed laparoscopic/endoscopic	Excision of Stomach, Via Natural or Artificial Opening Endoscopic, Diagnostic; Inspection of Stomach, Percutaneous Endoscopic Approach; Inspection of Lower Intestinal Tract, Percutaneous Endoscopic Approach; Release Small Intestine, Open Approach; Release Small Intestine, Percutaneous Endoscopic Approach; Release Peritoneum, Open Approach; Repair Stomach, Open Approach; Repair Stomach, Percutaneous Endoscopic Approach; Repair Stomach, Pylorus, Open Approach; Repair Stomach, Pylorus, Percutaneous Endoscopic Approach; Repair Small Intestine, Open Approach; Repair Small Intestine, Percutaneous Endoscopic Approach; Repair Duodenum, Open Approach; Repair Duodenum, Percutaneous Endoscopic Approach; Repair Jejunum, Open Approach; Repair Jejunum, Percutaneous Endoscopic Approach; Repair Transverse Colon, Open Approach; Repair Sigmoid Colon, Open Approach; Repair Sigmoid Colon, Percutaneous Endoscopic Approach; Supplement Stomach with Autologous Tissue Substitute, Open Approach; Supplement Stomach with Autologous Tissue Substitute, Percutaneous Endoscopic Approach; Supplement Stomach, Pylorus with Autologous Tissue Substitute, Open Approach; Supplement Stomach, Pylorus with Autologous Tissue Substitute, Percutaneous Endoscopic Approach; Supplement Duodenum with Autologous Tissue Substitute, Open Approach; Supplement Duodenum with Autologous Tissue Substitute, Percutaneous Endoscopic Approach; Supplement Duodenum with Autologous Tissue Substitute, Via Natural or Artificial Opening Endoscopic; Supplement Jejunum with Autologous Tissue Substitute, Open Approach; Supplement Jejunum with Autologous Tissue Substitute, Percutaneous Endoscopic Approach; Inspection of Peritoneal Cavity, Open Approach; Inspection of Peritoneal Cavity, Percutaneous Endoscopic Approach; Inspection of Gastrointestinal Tract, Open Approach; Repair Abdominal Wall, Open Approach; Irrigation of Peritoneal Cavity using Irrigating Substance, Percutaneous Approach;	0DU907Z, 0DQ90ZZ, 0DU947Z, 0DQ60ZZ, 0DQ70ZZ, 0DQ94ZZ, 0DQ64ZZ, 0DU607Z, 0DQA0ZZ, 0DQA4ZZ, 0WJP0ZZ, 0DU647Z, 0DB68ZX, 0DQ80ZZ, 0DU707Z, 0DUA47Z, 0DQN0ZZ, 0WQF0ZZ, 0DUA07Z, 0DN80ZZ, 0WJG0ZZ, 0WJG4ZZ, 0DQ84ZZ, 3E1M38Z, 0DQ74ZZ, 0DU747Z, 0DU987Z, 0DNW0ZZ, 0DQN4ZZ, 0DJ64ZZ, 0DJD4ZZ, 0DN84ZZ, 0DQL0ZZ	
	Temporizing surgery	Percutaneous Drainage/Diversion	Drainage of Stomach with Drainage Device, Via Natural or Artificial Opening; Drainage of Peritoneum with Drainage Device, Percutaneous Approach; Insertion of Feeding Device into Stomach, Open Approach; Insertion of Feeding Device into Stomach, Via Natural or Artificial Opening; Supplement Stomach with Synthetic Substitute, Open Approach; Supplement Duodenum with Synthetic Substitute, Open Approach; Drainage of Abdominal Wall with Drainage Device, Percutaneous Approach; Drainage of Peritoneal Cavity with Drainage Device, Open Approach; Drainage of Peritoneal Cavity, Open	0D9670Z, 0W9G30Z, 0DU90JZ, 0DU60JZ, 0DH60UZ, 0W9G00Z, 0W9G3ZZ, 0W9J30Z, 0W9G40Z, 0W9F30Z, 0DH67UZ, 0W9G3ZX, 0D9W30Z, 0W9G0ZZ	

 © 2023. The Author(s).

(*Continued*)

		Summary or general heading*	Shortened ICD 10 Text**	ICD10 codes	Mortality
			Approach; Drainage of Peritoneal Cavity with Drainage Device, Percutaneous Approach; Drainage of Peritoneal Cavity, Percutaneous Approach, Diagnostic; Drainage of Peritoneal Cavity, Percutaneous Approach; Drainage of Peritoneal Cavity with Drainage Device, Percutaneous Endoscopic Approach; Drainage of Pelvic Cavity with Drainage Device, Percutaneous Approach;		
	Difficult surgery - 1	Partial resections	Excision of Stomach, Open Approach, Diagnostic; Excision of Stomach, Open Approach; Excision of Stomach, Percutaneous Endoscopic Approach; Excision of Stomach, Pylorus, Open Approach; Excision of Stomach, Pylorus, Via Natural or Artificial Opening Endoscopic, Diagnostic; Excision of Small Intestine, Open Approach, Diagnostic; Excision of Small Intestine, Open Approach; Excision of Small Intestine, Percutaneous Endoscopic Approach; Excision of Duodenum, Open Approach; Excision of Duodenum, Via Natural or Artificial Opening Endoscopic, Diagnostic; Excision of Jejunum, Open Approach, Diagnostic; Excision of Jejunum, Open Approach; Excision of Jejunum, Percutaneous Endoscopic Approach; Excision of Ileum, Open Approach; Excision of Large Intestine, Open Approach; Excision of Right Large Intestine, Open Approach; Excision of Left Large Intestine, Open Approach; Excision of Cecum, Open Approach; Excision of Ascending Colon, Open Approach; Excision of Transverse Colon, Open Approach; Excision of Descending Colon, Open Approach; Excision of Sigmoid Colon, Open Approach; Excision of Sigmoid Colon, Percutaneous Endoscopic Approach; Excision of Rectum, Open Approach; Resection of Stomach, Pylorus, Open Approach; Resection of Small Intestine, Open Approach; Resection of Small Intestine, Percutaneous Endoscopic Approach; Resection of Jejunum, Open Approach; Resection of Jejunum, Percutaneous Endoscopic Approach; Resection of Ileum, Open Approach; Resection of Large Intestine, Open Approach; Resection of Right Large Intestine, Open Approach; Resection of Right Large Intestine, Percutaneous Endoscopic Approach; Resection of Left Large Intestine, Open Approach; Resection of Cecum, Open Approach; Resection of Appendix, Open Approach; Resection of Ascending Colon, Open Approach; Resection of Transverse Colon, Open Approach; Resection of Descending Colon, Open Approach; Resection of Sigmoid Colon, Open Approach; Resection of Sigmoid Colon, Percutaneous Endoscopic Approach; Resection of Gallbladder, Open Approach; Resection of Gallbladder, Percutaneous Endoscopic Approach;	0DB80ZZ, 0DBA0ZZ, 0DTN0ZZ, 0DTF0ZZ, 0DBN0ZZ, 0DB60ZZ, 0DBB0ZZ, 0DT80ZZ, 0DTG0ZZ, 0DTA0ZZ, 0DB90ZZ, 0DTH0ZZ, 0DB98ZX, 0DBL0ZZ, 0DB60ZX, 0DTE0ZZ, 0DB70ZZ, 0DTB0ZZ, 0DB84ZZ, 0DBH0ZZ, 0DB80ZX, 0DBA4ZZ, 0DBM0ZZ, 0DTN4ZZ, 0DBG0ZZ, 0DBN4ZZ, 0FT40ZZ, 0DT70ZZ, 0DTM0ZZ, 0DBF0ZZ, 0DT84ZZ, 0DBA0ZX, 0DTF4ZZ, 0DTL0ZZ, 0DTK0ZZ, 0DB78ZX, 0DB64ZZ, 0DTJ0ZZ, 0DBK0ZZ, 0DBP0ZZ, 0DBE0ZZ, 0DTA4ZZ, 0FT44ZZ	
	Difficult surgery - 2	Bypass performed	Bypass Stomach to Jejunum, Open Approach; Bypass Stomach to Jejunum, Percutaneous Endoscopic Approach; Bypass Ileum to Cutaneous, Open Approach; Bypass Transverse Colon to Cutaneous, Open Approach; Bypass Descending Colon to Cutaneous, Open Approach; Bypass Sigmoid Colon to Cutaneous, Open Approach; Bypass Sigmoid Colon to Cutaneous, Percutaneous Endoscopic Approach;	0D160ZA, 0D1B0Z4, 0D1N0Z4, 0D1L0Z4, 0D1M0Z4, 0D164ZA, 0D1N4Z4	

(*Continued*)

(*Continued*)

		Summary or general heading*	Shortened ICD 10 Text**	ICD10 codes	Mortality
Obstructions	Lowest mortality	No obvious pattern	Intestinal adhesions w obst (postprocedural) (postinfection); Intestnl adhesions, unsp as to partial versus complete obst; Intestinal adhesions [bands], with partial obstruction; Unspecified intestinal obstruction; Partial intestinal obstruction, unspecified as to cause; Unsp intestnl obst, unsp as to partial versus complete obst; Other intestinal obstruction; Other partial intestinal obstruction; Other intestnl obst unsp as to partial versus complete obst;	K5650, K5669, K56609, K56690, K565, K56699, K5660, K5651, K56600	1.55%
	Intermediate mortality	No obvious pattern	Obstruction of duodenum; Intestinal adhesions [bands] with complete obstruction; Complete intestinal obstruction, unspecified as to cause;	K5652, K56601, K315	3.61%
	Highest mortality	No obvious pattern	Other complete intestinal obstruction;	K56691	14.81%
	Least invasive surgery	Exploration, lysis of adhesions, minor resections	Inspection of Lower Intestinal Tract, Percutaneous Endoscopic Approach; Release Small Intestine, Percutaneous Endoscopic Approach; Release Jejunum, Percutaneous Endoscopic Approach; Release Ileum, Percutaneous Endoscopic Approach; Release Large Intestine, Percutaneous Endoscopic Approach; Release Cecum, Percutaneous Endoscopic Approach; Release Sigmoid Colon, Percutaneous Endoscopic Approach; Release Peritoneum, Percutaneous Endoscopic Approach; Resection of Appendix, Percutaneous Endoscopic Approach; Resection of Gallbladder, Percutaneous Endoscopic Approach; Inspection of Peritoneal Cavity, Percutaneous Endoscopic Approach; Inspection of Gastrointestinal Tract, Percutaneous Endoscopic Approach; Repair Abdominal Wall, Percutaneous Endoscopic Approach;	0DN84ZZ, 0DNB4ZZ, 0DNW4ZZ, 0WJP4ZZ, 0DNA4ZZ, 0WJG4ZZ, 0DJD4ZZ, 0DNN4ZZ, 0DNH4ZZ, 0FT44ZZ, 0DNE4ZZ, 0WQF4ZZ, 0DTJ4ZZ	
	Temporizing surgery	Percutaneous drainage, diversion, dilation	Dilation of Duodenum with Intraluminal Device, Via Natural or Artificial Opening Endoscopic; Dilation of Duodenum, Via Natural or Artificial Opening Endoscopic; Dilation of Sigmoid Colon with Intraluminal Device, Via Natural or Artificial Opening Endoscopic; Dilation of Sigmoid Colon, Via Natural or Artificial Opening Endoscopic; Drainage of Stomach with Drainage Device, Via Natural or Artificial Opening; Drainage of Stomach with Drainage Device, Via Natural or Artificial Opening Endoscopic; Drainage of Stomach, Pylorus with Drainage Device, Via Natural or Artificial Opening; Excision of Rectum, Via Natural or Artificial Opening Endoscopic, Diagnostic; Insertion of Feeding Device into Stomach, Percutaneous Approach; Insertion of Infusion Device into Stomach, Via Natural or Artificial Opening; Insertion of Feeding Device into Stomach, Via Natural or Artificial Opening; Inspection of Upper Intestinal Tract, Via Natural or Artificial Opening Endoscopic; Inspection of Lower Intestinal Tract, Via Natural or Artificial Opening Endoscopic; Drainage of Bladder with Drainage Device, Via Natural or Artificial Opening; Drainage of Peritoneal Cavity with Drainage Device, Percutaneous Approach; Drainage of Peritoneal Cavity, Percutaneous Approach, Diagnostic; Drainage of Peritoneal Cavity, Percutaneous Approach;	0D9670Z, 0DJD8ZZ, 0DJ08ZZ, 0DH67UZ, 0DH63UZ, 0W9G3ZZ, 0D798DZ, 0W9G3ZX, 0D798ZZ, 0D9680Z, 0T9B70Z, 0D9770Z, 0DBP8ZX, 0D7N8DZ, 0D7N8ZZ, 0W9G30Z, 0DH673Z	
	Difficult surgery - 1	Bowel bypass or repairs, open bowel releases	Bypass Stomach to Jejunum, Open Approach; Bypass Stomach to Jejunum, Percutaneous Endoscopic Approach; Bypass Ileum to Cutaneous, Open Approach; Bypass Ileum to Cutaneous, Percutaneous Endoscopic Approach; Bypass Transverse Colon to Cutaneous, Open Approach; Bypass Descending Colon to Cutaneous, Open Approach;	0DN80ZZ, 0DNB0ZZ, 0DNW0ZZ, 0DNA0ZZ, 0D1L0Z4, 0DQ80ZZ, 0D1B0Z4, 0WJP0ZZ, 0DNE0ZZ, 0D160ZA,	

 © 2023. The Author(s).

(*Continued*)

		Summary or general heading*	Shortened ICD 10 Text**	ICD10 codes	Mortality
			Bypass Sigmoid Colon to Cutaneous, Open Approach;; Release Small Intestine, Open Approach; Release Duodenum, Open Approach; Release Jejunum, Open Approach; Release Ileum, Open Approach; Release Ileocecal Valve, Open Approach; Release Large Intestine, Open Approach; Release Cecum, Open Approach; Release Transverse Colon, Open Approach; Release Sigmoid Colon, Open Approach; Release Omentum, Open Approach; Release Peritoneum, Open Approach; Repair Small Intestine, Open Approach; Resection of Left Large Intestine, Open Approach; Resection of Appendix, Open Approach; Inspection of Peritoneal Cavity, Open Approach; Inspection of Gastrointestinal Tract, Open Approach; Repair Abdominal Wall, Open Approach; Supplement Abdominal Wall with Synthetic Substitute, Open Approach;	0WQF0ZZ, 0D1N0Z4, 0WJG0ZZ, 0DNN0ZZ, 0DTG0ZZ, 0DNH0ZZ, 0DTJ0ZZ, 0WUF0JZ, 0D1M0Z4, 0DNL0ZZ, 0D164ZA, 0DNU0ZZ, 0DN90ZZ, 0DNC0ZZ, 0D1B4Z4	
	Difficult surgery - 2	More extensive resections	Excision of Small Intestine, Open Approach, Diagnostic; Excision of Small Intestine, Open Approach; Excision of Small Intestine, Percutaneous Endoscopic Approach; Excision of Jejunum, Open Approach; Excision of Ileum, Open Approach; Excision of Ileum, Via Natural or Artificial Opening Endoscopic, Diagnostic; Excision of Large Intestine, Open Approach; Excision of Large Intestine, Via Natural or Artificial Opening Endoscopic, Diagnostic; Excision of Cecum, Open Approach; Excision of Descending Colon, Open Approach; Excision of Sigmoid Colon, Open Approach; Excision of Sigmoid Colon, Percutaneous Endoscopic Approach; Excision of Sigmoid Colon, Via Natural or Artificial Opening Endoscopic, Diagnostic; Excision of Stomach, Via Natural or Artificial Opening Endoscopic, Diagnostic; Resection of Small Intestine, Open Approach; Resection of Small Intestine, Percutaneous Endoscopic Approach; Resection of Jejunum, Open Approach; Resection of Ileum, Open Approach; Resection of Large Intestine, Open Approach; Resection of Right Large Intestine, Open Approach; Resection of Right Large Intestine, Percutaneous Endoscopic Approach; Resection of Cecum, Open Approach; Resection of Sigmoid Colon, Open Approach; Resection of Sigmoid Colon, Percutaneous Endoscopic Approach;	0DB80ZZ, 0DBB0ZZ, 0DT80ZZ, 0DB68ZX, 0DTN0ZZ, 0DBA0ZZ, 0DBN0ZZ, 0DTF0ZZ, 0DB84ZZ, 0DBN8ZX, 0DTN4ZZ, 0DTH0ZZ, 0DBB8ZX, 0DTB0ZZ, 0DB80ZX, 0DBN4ZZ, 0DT84ZZ, 0DBH0ZZ, 0DTA0ZZ, 0DBE8ZX, 0DTE0ZZ, 0DTF4ZZ, 0DBM0ZZ, 0DBE0ZZ	

Note: Procedure codes: "2017 ICD-10 PCS codes file." Centers for Medicare and Medicaid Services. Accessed 12/12/22. URL: https://www.cms.gov/medicare/coding-billing/icd-10-codes/2017-icd-10-pcs-gem.
*Mortality groups were developed empirically based on observed mortality by diagnosis; the grouped codes do not always fall under one concept, but a general heading is provided where a pattern is present. Surgical procedure codes were examined by diagnosis and those that were performed on more than 1/1000 of patients with the diagnosis were grouped into general conceptual headings given above. Subjects with more than one procedure were classified according to the most invasive procedure performed.
**Diagnostic codes: "2017 Code Descriptions in Tabular Order." Centers for Medicare and Medicaid Services. Accessed 12/12/22. URL: https://www.cms.gov/medicare/coding-billing/icd-10-codes/2017-icd-10-cm-gem.

 © 2023. The Author(s).
